# Acemannan Used as an Implantable Biomaterial for Vital Pulp Therapy of Immature Permanent Teeth Induced Continued Root Formation

**DOI:** 10.3390/pharmaceutics12070644

**Published:** 2020-07-08

**Authors:** Tien Thuy Vu, Minh Truong Nguyen, Polkit Sangvanich, Quang Ngoc Nguyen, Pasutha Thunyakitpisal

**Affiliations:** 1 Dental Biomaterials Science Program, Graduate School, Chulalongkorn University, Bangkok 10330, Thailand; Vu.Th@student.chula.ac.th; 2 Department of Oral and Maxillofacial Surgery, School of Odonto-Stomatology, Hanoi Medical University, Hanoi 100000, Vietnam; nguyentruongminh@hmu.edu.vn; 3 Department of Chemistry, Faculty of Science, Chulalongkorn University, Bangkok 10330, Thailand; polkit.S@chula.ac.th; 4 Department of Communications and Computer Engineering, Faculty of Science and Engineering, Waseda University, Tokyo 169-0051, Japan; quang.nguyen@aoni.waseda.jp; 5 Research Unit of Herbal Medicine, Biomaterial and Material for Dental Treatment, Department of Anatomy, Faculty of Dentistry, Chulalongkorn University, Bangkok 10330, Thailand

**Keywords:** tissue regeneration, acemannan, vital pulp therapy, biomaterial, implantable material

## Abstract

Direct pulp-capping, a vital pulp therapy, is used to protect and preserve pulp vitality by applying a biomaterial on the pulp exposure site. Acemannan, a polysaccharide extracted from *Aloe vera*, induces osteodentin-bridge formation to cover the exposure site in vivo. We evaluated the effect of acemannan sponges on partial pulpotomized permanent teeth with caries or accident-induced pulp exposure (*n* = 50). After removing infected dentin and inflamed pulp tissue, the teeth were randomly divided into acemannan or control (mineral trioxide aggregate (MTA) groups (*n* = 25). The teeth were examined immediately after treatment (baseline) and at 6- and 12-month follow-ups for clinical and cone beam computed tomography (CBCT) examination. The three-dimensional tooth length and root apex area were simulated to determine treatment success. We found that the overall success rate in the acemannan and MTA groups from baseline to 12-month follow-up was 90.91% and 95.65%, respectively, with no significant difference between the two groups (*p* > 0.05). In the success teeth in both groups, the *root* length increased, and the apex area significantly decreased (*p* < 0.05), indicating continued root formation. Our results suggest that acemannan is a promising low-cost biomaterial for partial pulpotomy treatment for immature permanent teeth requiring vital pulp therapy.

## 1. Introduction

Dental caries is a major oral health problem, especially in developing countries. When the pulp is exposed due to caries, trauma, or mechanical excavation, the appropriate treatment, i.e., vital pulp therapy or root canal treatment, has to be determined. However, for immature permanent teeth (incomplete root formation with wide-open apical foramen), root canal treatment is not the preferred choice. Without a vital pulp, root formation will not proceed because, in these situations, the tooth will stop erupting and the root will be thin and unable to tolerate masticatory forces, thus, the tooth will be prone to fracture. Based on the American Academy of Pediatric Dentistry (AAPD) guidelines, caries- and traumatic injury-teeth clinically diagnosed with a normal pulp (symptom-free and normal response to vitality testing) or reversible pulpitis (vital pulp capable of healing) should be treated using vital pulp procedures [1]. Partial pulpotomy, a vital pulp therapy treatment, is generally regarded as the treatment of choice for immature teeth with exposed pulp tissue [2]. In performing partial pulpotomy, only the superficial inflamed coronal pulp tissue is removed, and the healthy remaining pulp tissue is covered with an appropriate material to promote pulp healing and continued root development (apexogenesis). Currently, mineral trioxide aggregate (MTA) is widely used as the material of choice for pulp capping [2]. However, this material has several disadvantages, such as difficult handling, long setting time [3], and high cost for developing countries [4]. Thus, many biomaterials have been proposed to induce complete root formation and preserve pulp vitality at the same time. Due to direct contact with vital tissue, capping biomaterials must be biocompatible, implantable, and demonstrate a regenerative effect in vitro and in vivo; especially in a clinical trial compared with a suitable control material as the definitive study.

Acemannan, a natural polysaccharide extracted from *Aloe vera*, has many medicinal properties, e.g., cell proliferation, osteogenic, immunomodulation, and antimicrobial, that promote wound healing [5,6,7,8]. In dentistry, acemannan induces pulp healing and dentin formation in vitro and in vivo [9,10]. In previous studies, we also demonstrated that acemannan upregulates growth factor expression and extracellular matrix synthesis, which play essential roles in mineral deposition [11,12]. Hence, acemannan could be a promising biological and economical alternative material to MTA when performing a partial pulpotomy. The aim of this study was to verify the effectiveness of acemannan by comparing its impact to that of MTA on partial pulpotomy in immature permanent teeth using a three-dimensional (3D) approach with cone beam computed tomogramphy (CBCT). 

## 2. Materials and Methods

### 2.1. Acemannan Sponge Preparation

Acemannan was extracted and characterized as previously described [13]. Briefly, *Aloe vera* pulp gel (*Aloe barbadensis* Miller) was obtained from mature leaves. After homogenization, centrifugation, precipitation with alcohol, and lyophilization, the white acemannan precipitate was characterized by 1H-NMR, 13C-NMR (Fourier 300, Bruker, Ettlingen, Baden-Württemberg, Germany), and FT-IR (Spectrum System 2000; PerkinElmer, Waltham, MA, USA) [14].

The isolated acemannan was used to prepare a 1% (*w*/*v*) acemannan solution. To produce the acemannan sponges, 0.1 mL acemannan solution was poured into a 5 × 5 × 4 mm^3^ stainless steel mold, frozen at −80 °C overnight, and then lyophilized for 16 h, generating 1-mg acemannan sponges. Thus, the sponges were composed solely of acemannan. The acemannan sponges were sent to the Thailand Institute of Nuclear Technology, Bangkok, Thailand, for sterilization using gamma irradiation. The sponges were kept in a desiccator at room temperature until used.

### 2.2. Study Design

A non-inferiority clinical trial was conducted to compare the effectiveness of ProRoot MTA (control group; Dentsply, Tulsa, OK, USA) and acemannan sponges (test group) as capping materials in partial pulpotomized immature permanent teeth.

### 2.3. Study Participants

Patients (age range 7–13, mean 9.2 ± 1.5 years old) were recruited from the Department of Restorative and Endodontics and the Emergency Department, National Hospital of Odonto-stomatology, Hanoi, Vietnam between October 2015 and October 2017. The immature permanent tooth eruption stage was determined by the patient’s age and radiographic evaluation before enrolling in the research study. The inclusion criteria were an immature permanent tooth with a deep carious lesion that led to a caries-exposed pulp after spoon excavation and a diagnosis of reversible pulpitis, or the tooth had a pulp exposure caused by trauma that had occurred within 48 h.

The exclusion criteria were:

1. The patient had a history of systemic disease that interferes with pulp healing.
2. The tooth had clinical signs and symptoms of irreversible pulp pulpitis, e.g., spontaneous throbbing pain, tenderness to percussion, abnormal tooth mobility, swelling, or sinus tract.
3. Radiographic evidence of internal or external resorption, inter-radicular bone loss, or periapical pathology.
4. The tooth was non-restorable.

During the study, if any symptoms of irreversible pulpitis, apical periodontitis, or infection occurred, the patients received the appropriate treatment following the institute protocol, including regenerative and root canal treatment [4].

### 2.4. Research Ethics

The study protocol was approved by the Ethics Committee (No. 217/HDDD-BVRHMTW, approval date: 22 July 2015), and registered in the Thai Clinical Trials Registry (registration number TCTR20190923004). The study details were explained to the children and their parents, and they signed consent forms before starting any clinical procedure.

### 2.5. Research Sample Size

The sample size was estimated using the formula of Bouman et al. [15]. The success rate of MTA in partial pulpotomy in immature permanent molars was 94.4% [16], while the success rate of acemannan in partial pulpotomy immature permanent molars has not been reported. Therefore, the success rate for acemannan was assumed to be not less than that of MTA. Using a 20% non-inferiority limit, 80% test precision, and 5% type I error, the required sample size was calculated and 18 teeth per material group were required (the detailed formula and calculation are presented in Appendix A). Considering a 20% drop-out from the study, the sample size was required to be at least 22 teeth in each group.

### 2.6. Study Intervention

This was a randomized, controlled, single-blinded clinical trial. The teeth were randomly assigned to the groups using a randomized table with a block size of 4 by a clinical coordinator, i.e., the principal dentist did not decide which teeth went into which group. Instead, the dentist only selected the cases appropriate for partial pulpotomy. The patients were also blinded to their material groups. The teeth were treated by an unblinded endodontist who performed the treatment strictly adhering to the AAPD guidelines [1]. The CBCT was performed using an ultralow dose, small field of view, and appropriate radiation protection as described by the American Association of Endodontics (AAE) and the American Academy of Oral and Maxillofacial Radiology (AAOMR), and the European Society of Endodontology guidelines [17,18].

Each tooth was infiltrated with local anesthesia using 4% articaine with 1:100,000 epinephrine (Septanest, Septodont, France). The tooth was isolated with a rubber dam and washed with 0.12% chlorhexidine. The cavity was prepared, and the infected enamel and dentin were removed with a round bur. The carious dentin close to the pulp was removed using a spoon excavator. The pulp exposure position was identified, and the inflamed pulp tissue (1.5–2 mm) was gently removed using an abrasive diamond bur at high speed with copious sterilized normal saline. The remaining healthy pulp was rinsed with 2.5% sodium hypochlorite, and moist, sterilized cotton pellets were placed over the pulp stumps with light pressure for 2 min to achieve hemostasis. If the bleeding could not be controlled, the tooth was excluded from this study [1].

MTA or acemannan was then placed over the remaining healthy pulp per the manufacturer’s recommendation. To cover the exposure area, MTA and acemannan sponge were placed using an amalgam carrier and non-toothed Adson forceps, respectively. When required, the acemannan sponge was gently pressed with finger-force to adjust its shape to fit the exposure site prior to placing it. The cavities were lined with glass ionomer cement (Vitrebond, 3M ESPE, St. Paul, MN, USA), and a permanent composite restoration (Filtex Z350, 3M ESPE, Salt Lake City, UT, USA) was placed. If a traumatized tooth was classified as grade 1 or 2 tooth mobility, following the AAPD guidelines, a composite splint was applied for stabilization and removed 4–8 weeks post-surgery [19]. After treatment, a periapical radiograph and CBCT (ProMax 3D; Planmeca, Helsinki, Finland) were immediately taken with ultra-low dose mode (90 kVp, 5.6 mA, 4 × 5 cm FOV, and 4 s) to serve as the baseline with a reduced dose, due to the need for future follow-ups. Shielding devices, a leaded thyroid collar, leaded glasses, and a leaded apron, were used to protect the patient’s thyroid gland, eye lens, body, respectively [20].

### 2.7. The Follow-Up Examination Procedure

The patients and their parents were interviewed by telephone on day 1 and day 7 post-surgery about any pain or adverse reactions related to the treatment. Paracetamol was prescribed if the patients had pain, however, no antibiotic was used following the surgery. The patients received a 3-month follow-up clinical and periapical radiographic examination, together with 6- and 12-month clinical and CBCT examinations.

The clinical and radiograph evaluations were performed by two blinded experienced endodontists. The examiners were trained in the evaluation criteria prior to the study. To determine the reliability of the evaluations, Kappa scores were determined by the 2 examiners re-evaluating all cases 1 month after the initial evaluation was performed. The intra- and inter-examiner Kappa scores of the clinical evaluation were 1.0. For the radiographic evaluation, the intra-rater and inter-rater reliabilities were 0.91 and 0.89, respectively. When there was a disagreement, both examiners discussed the clinical and radiographic findings to achieve a consensus.

### 2.8. 3D Tooth Reconstruction and Analysis of the Partial Pulpotomy Treatment

**Figure 1:**
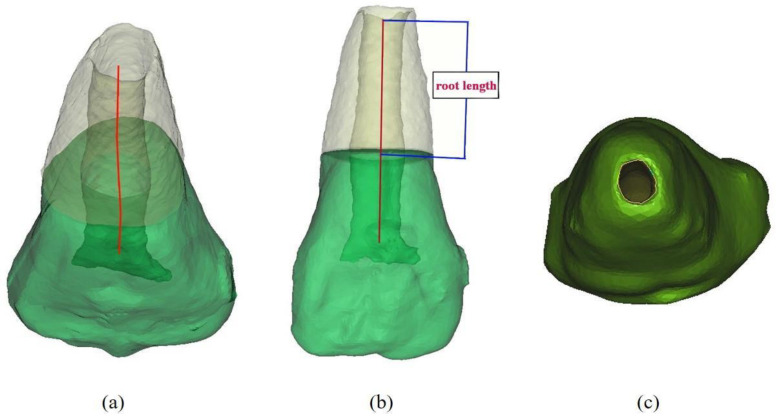
Measurement of the root length and apical foramen area of the 3D reconstructed tooth. (**a**) the center line (CT line) was created in the center of the root canal. The cementoenamel junction was generated as the horizontal plane (CE plane); (**b**) the root length was determined from the intersection of the center line and the horizontal plane to the apical foramen; (**c**) apical view of the tooth. The apical foramen was outlined, and its area was calculated.

The CBCT data were used to construct the 3D tooth images using Mimics software (Materialise, Leuven, Belgium). A center line (CT line) was created through the center of each root canal (Figure 1). The cementoenamel junction was identified, and the lowest points of the labial/buccal and lingual/palatal cementoenamel junction were used to generate the horizontal plane (CE plane). The length of each root was determined from the intersection of the CT line and the CE plane to the apical foramen (Figure 1a,b). Using the apical view, the apical foramen was located, and the apical foramen area was measured (Figure 1c). The measurements were performed three times, and the mean value was recorded. If the tooth had more than one root, the mean root length and the mean apical foramen area were calculated to evaluate the changes in those measurements immediately, 6-, and 12-months post-surgery. The 3D reconstruction and analysis were performed by the same blinded experienced radiologist. Each CBCT image was evaluated twice, with a 1-month break between evaluations and the intra-operator reliability for CBCT measurements was high (Pearson correlation coefficient, 0.92). The superimposition of the immediate, 6-, and 12-months post-surgery 3D tooth images was performed using the 3-Matic software (Materialise, Leuven, Belgium).

### 2.9. Criteria for the Evaluation of Overall Research Treatment

**Figure A1:**
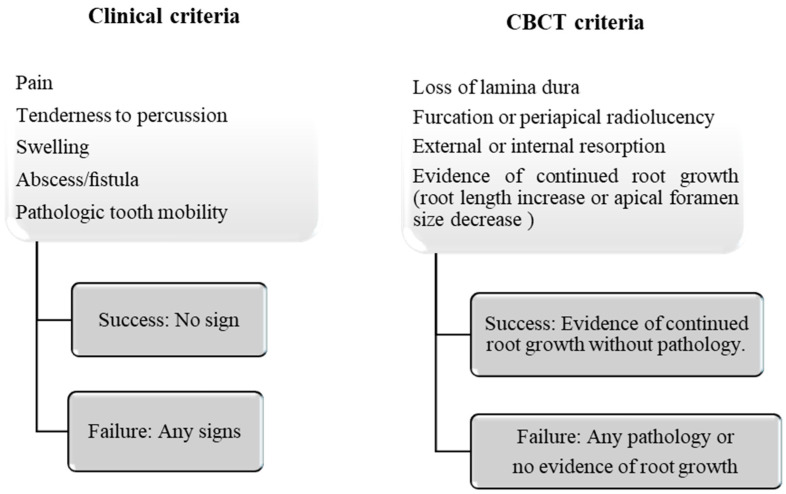
Outcome evaluation criteria.

The overall treatment outcome was determined using the clinical and CBCT assessments. The criteria for clinical failure were negative to cold, presence of signs or symptoms of reversible or irreversible pulpitis, pain on percussion, swelling, pus exudates/fistulae in the soft and periodontal tissues, or abnormal tooth mobility. The criteria for radiographic failure were based on loss of the lamina dura, discontinued root formation, or a more advanced periapical lesion. To confirm the continued root formation, the success cases had to demonstrate evidence of increased root length or decreased apical foramen area based on 3D measurements. If the treated tooth had any clinical or radiographic failure criteria, it was defined as a failure. The outcome evaluation criteria for success/failure of the clinical and CBCT-based methods are presented in Figure A1.

### 2.10. Statistical Analyses

Statistical analyses were performed using SPSS statistical software (SPSS Science, Chicago, IL, USA). The clinical and radiographic outcomes and overall outcomes between the MTA and acemannan groups were evaluated and compared using the chi-squared test. The mean root length and apical foramen area of each group at the baseline, 6- and 12-month follow-up were calculated and compared using one-way ANOVA. A *p*-value less than 0.05 was considered significant.

## 3. Results

### 3.1. Overall Clinical and Radiographic Success of Acemannan Treatment

#### 3.1.1. Acemannan Demonstrated Similar Overall Clinical and Radiographic Success Rates to Those of MTA

**Figure 2:**
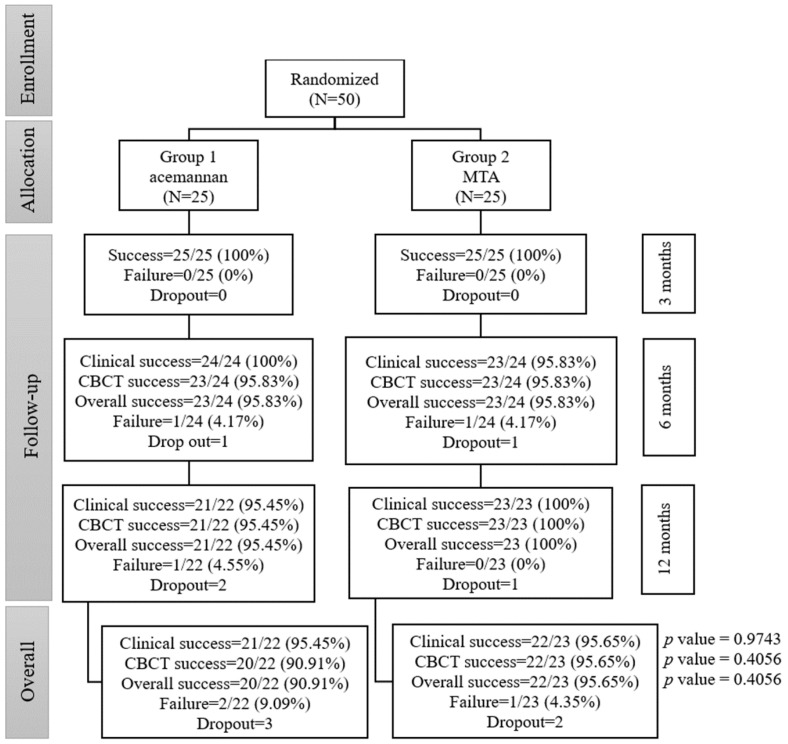
The flow of the participants through each stage of the study. The differences in the overall clinical and radiographic success rates with clinical evaluation and cone beam computed tomography (CBCT) data between the acemannan and mineral trioxide aggregate (MTA) groups were analyzed using the chi-squared test.

Fifty permanent teeth (23 teeth with trauma exposures and 27 teeth with caries exposures) were used in this study. The teeth were randomly divided into either the acemannan group (25 teeth: 12 trauma and 13 caries) or the MTA group (25 teeth: 11 trauma and 14 caries). At the 6-month follow-up period, 48/50 teeth (24/25 teeth in the acemannan group and 24/25 teeth in the MTA group) were available for clinical and radiographic evaluation. At the 12-month follow-up, 45/48 teeth (22/24 teeth in the acemannan group and 23/24 teeth in the MTA group) were available for clinical and radiographic evaluation. Five teeth (three acemannan, two MTA) were lost to follow-up, because the patients’ families moved out of the area during the study period. A flowchart of the participants in each stage of the trial is illustrated in Figure 2.

The results of the post-treatment telephone interviews indicated that three patients experienced mild pain on day 1. After prescribing paracetamol for pain relief, neither pain nor any adverse effects were reported. The patients with traumatic exposures were re-appointed to evaluate at 4- and 8-weeks post-surgery. The composite splints were removed when the patients passed the evaluation.

At the 6-month follow-up, one tooth (trauma case) in the MTA group was diagnosed with irreversible pulpitis, due to sharp pain and positive for percussion and a widened periodontal space. One tooth (trauma case) in the acemannan group was asymptomatic; however, the radiographic evaluation indicated a radiolucent area at the tooth apex. The clinical success rate, radiographic success rate, and overall success rate in the acemannan group was 100% (24/24), 95.83% (23/24), and 95.83% (23/24), respectively. Furthermore, the clinical success rate, radiographic success rate, and overall success rate in the MTA group was 95.83% (23/24), 95.83% (23/24), and 95.83% (23/24), respectively (Figure 2). These results demonstrated that there was no significant difference between the overall success rates of the acemannan and MTA groups at 6-months post-treatment (*p* > 0.05).

At 12-months post-treatment (from 6 to 12 months), one tooth (caries case) in the acemannan group was diagnosed with irreversible pulpitis, due to sharp pain and positive for percussion and radiographic evidence of a discontinuous lamina dura of the root. The clinical success rate, radiographic success rate, and overall success rate in the acemannan group was 95.45% (21/22), 95.45% (21/22), and 95.45% (21/22), respectively (Figure 2). Similarly, the clinical success rate, radiographic success rate, and overall success rate in the MTA group was 100% (23/23), 100% (23/23), and 100% (23/23), respectively. Moreover, there was no significant difference between the overall success rates of the acemannan and MTA groups at 12-months post-treatment (*p* > 0.05) (Figure 2).

Overall, the total success rates in the acemannan and MTA groups from baseline to 12-month follow-up were 90.91% (20/22) and 95.65% (22/23), respectively (Figure 2). There was no significant difference between the overall success rates in the acemannan and MTA groups from baseline to the 12-month post-surgery follow-up (*p* > 0.05). The difference between the total overall success rate of each group was −4.74%, and the 95% confidence interval (CI) was −19.36% to 9.88%. The lower limit of the 95% CI for the difference between the acemannan and MTA groups was above the inferiority limit of −20%. Therefore, acemannan was statistically non-inferior to MTA.

#### 3.1.2. Acemannan Induced Continued Root Formation

**Figure 3:**
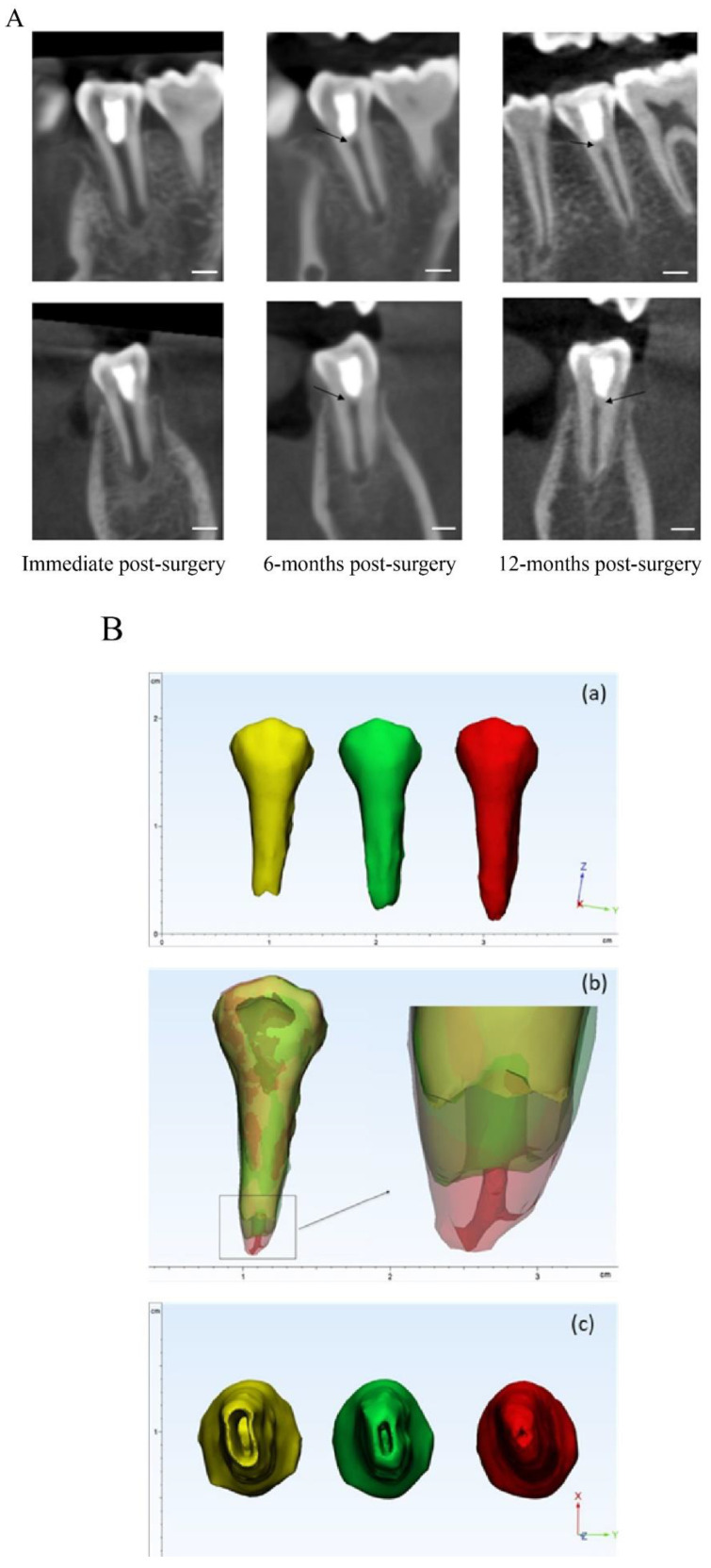
Representative images of an MTA treated tooth; (**A**): radiographic image in the sagittal and coronal views of the tooth immediately post-surgery, 6-months post-surgery, and 12-months post-surgery. Dentin bridge (black arrow) and continued root formation were observed at 6- and 12-months follow-up in a time-dependent manner. (**B)**: (**a**) 3D reconstructed tooth immediately post-surgery (yellow), 6-months post-surgery (green), and 12-months post-surgery (red). Increased root development post-treatment was observed; (**b**) the superimposed 3D tooth immediately, 6-months, and 12-months post-surgery. The enlarged image demonstrates the root apex position and root canal shape of the tooth; (**c**) the apical view of the tooth at the 3 time points. The narrowing of the apical foramen is demonstrated in a time-dependent manner. Scale bar = 3 mm.

**Figure 4:**
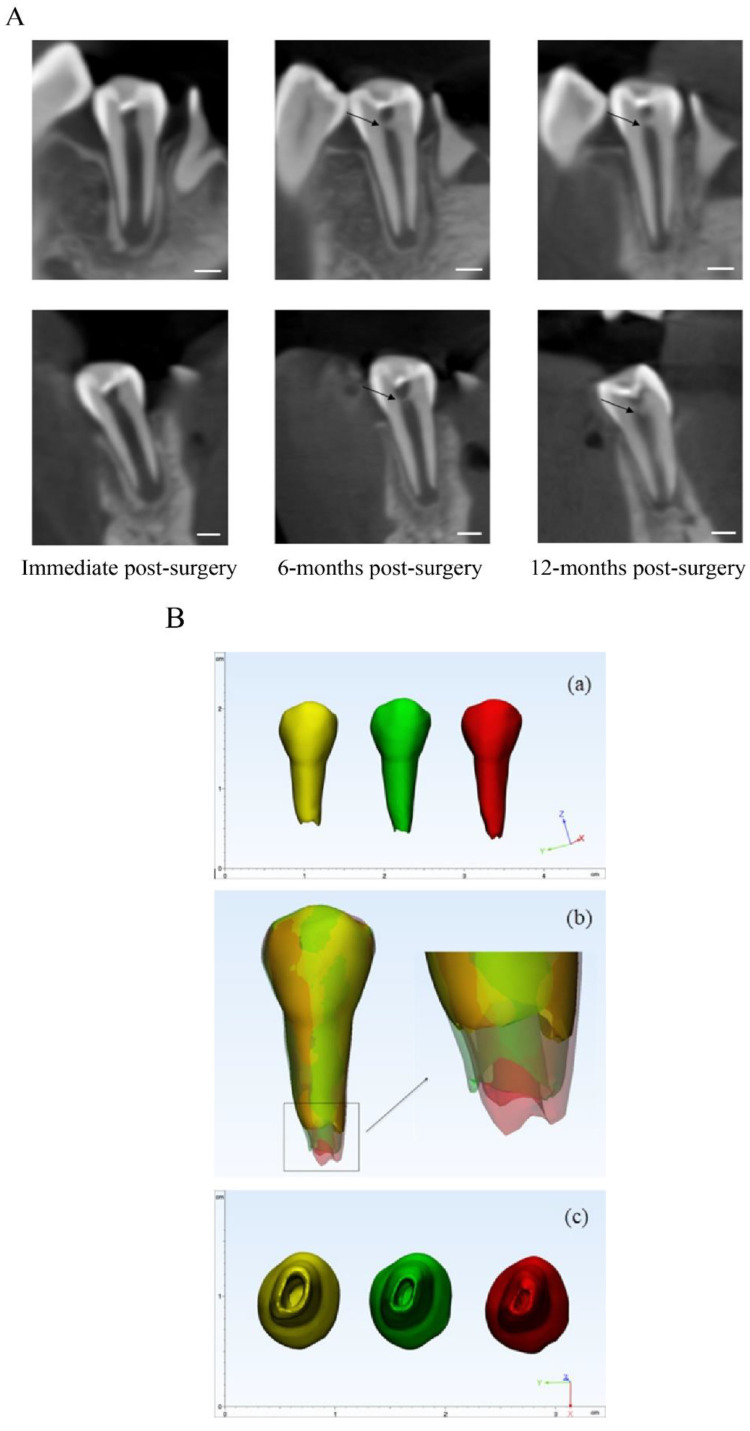
Representative images of an acemannan treated tooth; (**A**): radiograph image in the sagittal and coronal views of the tooth immediately post-surgery, 6-months post-surgery, and 12-months post-surgery. Dentine bridge (black arrow) and continued root formation were observed at 6- and 12-months follow-up in a time-dependent manner. (**B**): (**a**) 3D reconstructed tooth immediately post-surgery (yellow), 6-months post-surgery (green), and 12-months post-surgery (red). Increased root development post-treatment was observed; (**b**) the superimposed 3D tooth immediately, 6-months, and 12-months post-surgery. The enlarged image demonstrates the root apex position and root canal shape of the tooth; (**c**) the apical view of the tooth in 3 time points. The narrowing of the apical foramen is demonstrated in a time-dependent manner. Scale bar = 3 mm.

The mean radiographic root length and apical foramen area in the acemannan and MTA groups at baseline, 6- and 12-months post-treatment are shown in Table 1. The percentage increase in the mean radiographic root length in the acemannan group at 6- and 12-months post-surgery was 5.81 and 10.82, compared with the baseline value, respectively, and those in the MTA group were 7.72 and 11.98, respectively. A significant increase in the root length in each group was observed at 6- and 12-months post-surgery compared with the baseline value (*p* < 0.05, Table 1). Moreover, the apical foramen area in both groups demonstrated a significant decrease at 6- and 12-months follow-up (*p* < 0.05; Table 1). These data demonstrated that MTA and acemannan treatment successfully preserved pulp vitality and continued root formation. Thus, the MTA and acemannan groups had the same tendency for the partial pulpotomy treatment outcome, and there was no significant difference in the root length or apical foramen area between the two groups at the baseline and follow-up evaluations. Representative radiographs and superimposed tooth image, from the MTA and acemannan groups demonstrating the continued increase in the root length, dentin bridge formation, and decreased apical foramen area are presented in Figure 3 and Figure 4, respectively.

## 4. Discussion

Biomaterials are an important component of cell-free tissue engineering. These materials are designed to provide an architectural scaffold reminiscent of the native extracellular matrix, and may function like growth factors to enhance cell attachment and function, and eventually tissue regeneration. Apexogenesis is the primary goal of vital pulp treatment of immature permanent teeth with open apices for completing root apex formation [1]. To achieve efficient apexogenesis, partial pulpotomy is considered the appropriate treatment for an immature permanent tooth with traumatic or caries-induced pulp exposure, due to its benefits compared with root canal treatment and conventional pulpotomy [21]. This method preserves the pulp and periapical tissue, and maintains its function in continuing root development and the dentin formation, resulting in a reduced risk of crown fracture [22].

Currently, MTA is used as the pulp regenerative material of choice, due to its biocompatibility and osteoconductive properties [23]. MTA has favorable physiochemical characteristics: hermetic seal, bacterial penetration resistance, antimicrobial activity, and biocompatibility [24]. MTA provides an aseptic barrier and stimulates reparative dentinogenesis by recruiting and stimulating hard tissue-forming cells, and contributing to matrix formation and mineralization [25]. MTA also stimulates reparative hard tissue formation by sequestering growth factors and cytokines embedded in the surrounding dentin matrix [26]. The success rate (95%) of MTA in our study corresponds to those in previous studies that report the success rate of MTA in carious- and traumatized-exposure permanent teeth with pulp exposure from 93–100% [4,16,27,28,29]. Bogen et al. reported a 98% success rate in immature and mature permanent teeth (49 of 53 teeth) at 9 years of observation, and all immature teeth (15/15) demonstrated completed root formation [29].

Acemannan is a beta-1,4-polymonnose extracted from *Aloe vera* parenchyma. 1H- and 13C-nuclear magnetic resonance spectroscopy, gas chromatograph-mass spectrometer, and high performance liquid chromatography analysis revealed that acemannan was mainly composed of mannose (57–77%), glucose (15–22%), and galactose (5–7%), forming a chain of repeating tetrasaccharide units: -*o*-(acetyl mannose)-*o*-(acetyl mannose)-*o*-(glucose)-*o*-(acetyl mannose) with a single-branched galactose at the second or fourth acetylated mannose residues [8,30,31,32]. The average molecular weight of acemannan ranged from 150–800 kDa, depending on the extraction technique [8]. The acetyl group located on the mannose residue has been proposed as an acemannan functional domain. The bioactivity of acemannan comes from its 3D intramolecular and intermolecular architecture rather than the building block monosaccharides [14,31]. Although the regenerative mechanism of acemannan has not been explicitly identified and analyzed, prior studies demonstrated that acemannan induced pulp healing and dentin formation in vitro and in vivo [10,13]. Acemannan also stimulates dentin matrix protein expression (e.g., type I collagen, osteopontin, dentine sialophosphoprotein, alkaline phosphatase), growth factor secretion (BMP-2, BMP-4, VEGF), and mineralization by dental pulp cells. In addition, acemannan has immunomodulatory activity. Selectively binding to TLR5, acemannan enhanced NF-κB DNA binding activity, and upregulated proinflammatory cytokine IL-6 and -8 expression [14]. Therefore, acemannan might hasten the progression from the inflammatory phase to the formative phase of tissue regeneration.

In vivo, acemannan induced pulp healing and reparative dentin formation in lipopolysaccharide-induced reversible pulpitis in canine teeth and caries-exposed reversible pulpitis in human primary teeth [9,10]. Similar to MTA treatment, the use of acemannan resulted in histological-mineralized bridge formation with normal underlying pulp tissue without inflammation or pulp necrosis [9]. In addition, acemannan functions as a 3D scaffold to enhance blood clot formation [10]. We observed that acemannan sponges absorb blood, forming a gel-like blood clot. The neighboring odontoblasts, pulpal fibroblasts, and progenitor stem cells in the pulp migrate to the scaffold and generate dentin formation. The radiographic dentin bridge formation covered the exposure site and apexpogenesis confirmed the regenerative activity of acemannan. Therefore, acemannan has the characteristics of a promising and practical capping material for an economical and efficient partial pulpotomy procedure. However, based on its monosaccharide composition, acemannan sponges are radiolucent and the portion initially in contact with the pulp will be absorbed. The CBCT images demonstrated that the dentin bridge sealing the pulp was located in the radiolucent area that is between the pulp and acemannan sponge. To prevent the misinterpretation of this as a void space or secondary caries, incorporating biocompatible a radiopaque inorganic filler, e.g., calcium phosphate or calcium silicate into the acemannan sponges to generate a hybrid organic-inorganic material that is radiopaque and would not be misinterpreted.

In this study, the electrical pulp test is not included in the evaluation criteria. Although this technique has a high specificity and positive predictive value, its sensitivity and reproducibility are quite low [33,34]. This test has been demonstrated to be unreliable and relatively ineffective in primary teeth and immature permanent teeth [35]. False positive responses can occur in anxious and emotional child patients, due to anticipating an unpleasant sensation. The immature teeth with incomplete root formation produce a false negative response, due to few myelinated nerve fibers in the plexus of Rashkow at the dentin-pulp border. Traumatized teeth also provide false negative results because of nerve rupture and ischemic injury.

CBCT is a radiographic modality that has been proven to be superior to traditional 2D radiographs. Due to its ability to accurately reproduce the periapical tissues and their three-dimensional relationship to anatomical landmarks, CBCT can be a powerful tool in endodontic diagnosis, treatment planning, and follow-ups when the imaging requirements cannot be achieved by lower dose two-dimensional radiographs [17,18,36,37]. With a shorter exposure time (4 sec) and no pain, the child patients cooperate well. However, the radiation dose in CBCT is higher, compared with periapical radiographs. In this study, the patients received CBCT examination three times within a year (baseline, 6- and 12-months post-operation), with an ultra-low dose mode for research proposes. For safety, the patients wore lead devices to protect their sensitive tissues; thyroid gland, eye lens, and chest organs from radiation [20].

To the best of our knowledge, there are few studies using a three-dimensional approach, using CBCT for outcome assessment and follow-ups in vital pulp therapy in general and partial pulpotomy specifically. Moreover, successful vital pulp treatment of immature permanent teeth should result in continued root development, as demonstrated by increased root length and increased dentin thickness [1,37]. In some circumstances, such as nearly complete root formation, apexogenesis can be demonstrated by a narrowing apical foramen. However, this marker has not been used often due to the limitation of 2D radiographs in observing the apical foramen. Thus, using CBCT as a research tool can overcome this technical difficulty. Using CBCT allows for the same alignment and observation angle of the tooth image at each evaluation time point. In addition, our approach using CBCT revealed that 100% of the successful MTA- and acemannan-treated teeth (42/42) had apical foramen reduction, while only 90.48% of the success cases (38/42) showed increased root length. These findings suggest that a reduced apical foramen area, evaluated via CBCT, can be used as a criterion for apexogenesis in a research study with the appropriate precautions, especially when an increased root length is undetectable.

Clinically, slight changes/errors in the image angulation obtained at different time points are unavoidable, resulting in inaccurate and distorted 3D images that can lead to errors in measurement, data interpretation, and comparison between samples [38,39]. In the present study, we used CBCT to superimpose the 3D tooth images (single, two, and multiple roots) at different observation time points to compare and evaluate continued root formation via the root length and apical foramen area. In addition, changes in root morphology, such as size and curvature, even with multiple roots, can be observed. Through this kind of integrated approach, the superimposed image can act as an efficient tool for clinicians to use to evaluate and educate the patient to understand the treatment outcome. Overall, using the 3D approach for tooth reconstruction design and analysis, our results indicate that acemannan can be used as an alternative pulp capping material for reversible pulpitis. Although the overall success rate of MTA (95%) was slightly higher compared with the acemannan sponges (91%) at the 12-month follow-up, the success rate of acemannan was not statistically inferior to MTA. However, the superimposed image construction and analysis have some disadvantages. Currently, the process of 3D image superimposition takes time, and requires special technical knowledge to perform. Moreover, the contrast and details of the tooth structure can be unclear in the superimposed image, due to color blending.

However, it should be noted that although CBCT may be more accurate to examine the root length, CBCT should not be used routinely for screening purposes or endodontic diagnosis in the absence of clinical signs or symptoms, and used only when the patient’s history and a clinical evaluation indicate that the benefits exceed the potential risk, on a case-by-case basic [17,18]. In this study, CBCT was selected and performed for only research purposes, which have more requirements in evaluation compared with standard treatment and follow-up protocols.

The limitation of this study is the restricted observation period of 12-months, which was relatively shorter than that recommended in some studies to evaluate long-term treatment success [40,41,42]. Tooth discoloration by MTA is a major disadvantage, especially in the esthetic anterior teeth area [43]. Futures studies should include an evaluation of tooth discoloration post-treatment in both materials. Radiographic pathologies, e.g., internal or external root resorption, pulp calcification, and periapical radiolucency, develop over the long-term, and are difficult to measure, especially for immature permanent teeth with a physiological periapical radiolucency [38]. In addition, the best method to evaluate pulp healing is histological analysis, which cannot be performed in a clinical study. The osteodentin bridge is considered a favorable response to vital pulp therapy, which requires histological examination to confirm the quality of this mineralized tissue. However, in some cases, continued root formation of immature permanent teeth receiving vital pulp therapy do not show the formation or increased thickness of osteodentin bridge formation in conventional radiographs [41]. Nowika et al. reported that CBCT identified 25 bridges out of the 37 confirmed by histopathology (67%). The similar average density of young dentin and an osteodentin bridge and the artifacts, due to the restorations make osteodentin bridge formation difficult to discern radiographically [42]. Currently, a study of the relation/correlation between 3D radiographic osteodentin bridge formation, increased osteodentin bridge thickness, and continued root formation in successful partial pulpotomized immature teeth over long-term observation is ongoing. In the present study, acemannan was extracted from *Aloe vera* at a laboratory scale, and commercially available acemannan was not used. To confirm the regenerative effect of acemannan, large scale clinical studies of this polysaccharide in vital pulp therapy should be performed. Setting standardized criteria for acemannan extracted from *Aloe vera* will support and accelerate the application of this natural biomaterial in tissue regeneration.

## 5. Conclusions

Based on clinical and CBCT-based evaluations, acemannan is a promising implantable biomaterial as a capping material for partial pulpotomy of immature permanent teeth to induce continued root formation. The novel proposed 3D superimposition of tooth images at different time points could be used as a reliable tool for evaluating the partial pulpotomy treatment of immature permanent teeth.

## Figures and Tables

**Table 1:** The 3D radiographic root length and apical foramen area of the acemannan and MTA groups at baseline, 6- and 12-months post-treatment. The differences in the root length and apical foramen area were analyzed using one-way ANOVA.

Groups	Observation Time	Root Length (mm)			Apical Foramen Area (mm^2^)		
		Mean	SD	SE	Mean	SD	SE
Acemannan (*n* = 20)	Immediate	10.163	1.917	0.336	2.632	2.979	0.535
	6 months	10.753	1.772	0.325 *	1.912	2.299	0.387 *
	12 months	11.263	1.525	0.292 *	1.137	1.367	0.235 *
MTA (*n* = 22)	Immediate	10.735	1.331	0.349	2.289	1.701	0.511
	6 months	11.565	1.092	0.310 *	1.288	0.954	0.369 *
	12 months	12.021	1.065	0.278 *	0.629	0.646	0.224 *

* Significant difference compared with its baseline (immediate obserbation time) value; *p* < 0.05.

## Appendix A

Sample size calculation formula.

$$nA=\kappa \;nB\;\mathrm{and}\;nB=\left(\frac{p_{A}\left(1-p_{A}\right)}{\kappa }=p_{B}\left(1-p_{B}\right)\right){\left(\frac{z_{1-\alpha }+z_{1-\beta }}{p_{A}-p_{B}-\delta }\right)}^{2}$$

$$1-\beta =\Phi \left(z-z_{1-\alpha /2}\right)+\Phi \left(-z-z_{1-\alpha /2}\right),\;z=\frac{p_{A}-p_{B}-\delta }{\sqrt{\frac{p_{A}\left(1-p_{A}\right)}{n_{A}}+\frac{p_{B}\left(1-p_{B}\right)}{n_{B}}}}$$

where

- *κ* = *nA*/*nB* is the ratio between the sample sizes of the two groups (*κ* = 1 means that each group has an equal sample size)
- *Φ* is the standard Normal distribution function
- *α* is a Type I error (*α* = 0.05)
- *β* is a Type II error, i.e., 1 − *β* is power (*β* = 0.2)
- *δ* is the testing non-inferiority margin (*δ* = 0.2)
